# Vaccine Science Diplomacy: Expanding Capacity to Prevent Emerging and Neglected Tropical Diseases Arising from Islamic State (IS)–Held Territories

**DOI:** 10.1371/journal.pntd.0003852

**Published:** 2015-09-24

**Authors:** Peter J. Hotez

**Affiliations:** 1 United States Science Envoy Program, Department of State, Washington, D. C., United States of America; 2 Sabin Vaccine Institute and Texas Children’s Hospital Center for Vaccine Development, National School of Tropical Medicine, Baylor College of Medicine, Houston, Texas, United States of America; 3 Department of Biology, Baylor University, Waco, Texas, United States of America; 4 James A. Baker III Institute of Public Policy, Rice University, Houston, Texas, United States of America; National Institutes of Health, UNITED STATES

## Introduction

War and the ensuing health system breakdowns in the Islamic State (IS)–occupied Syria and Iraq significantly increase the risk of a new wave of infectious disease epidemics in the Middle East and North Africa (MENA). Proactive engagement to enable health system capacity and resilience—including expanding immunization programs and building biotechnology capacity for vaccines that specifically target diseases in the region—would help minimize the impact if and when outbreaks occur. A program of vaccine science diplomacy with selected countries in the MENA region could help to avert an international public health crisis possibly similar in scope and magnitude to the 2014 Ebola virus outbreak in West Africa.

The 2014 Ebola outbreak emphasized strong links between the forces of poverty, depletions in public health and environmental degradations as a result of long-standing conflicts in West Africa, and the emergence of a catastrophic neglected tropical disease (NTD). A stark reality is that such links between poverty, war, and NTDs are not new, but have been reoccurring for decades [1].

For example, beginning in the 1970s and lasting throughout much of the 20th century, hundreds of thousands of people may have perished from African sleeping sickness—human African trypanosomiasis, a parasitic infection transmitted by tsetse flies—in Angola, Democratic Republic of Congo, and Sudan because of civil wars in those countries and the inability to mount effective public health control measures [2]. Kala-azar—visceral leishmaniasis, another parasitic infection but transmitted by sandflies—killed an estimated 100,000 people in conflict-ridden southern Sudan between 1986 and 1995 [3]. Because journalists had limited access to these war-torn areas, both epidemics went mostly unrecorded and unacknowledged. The latest example is the collapsed health systems of post-conflict Liberia and Sierra Leone that were unable to cope with an Ebola epidemic that infected more than 20,000 people and caused approximately 10,000 deaths by the early part of 2015.

## Neglected Diseases and Emerging Infections in the MENA

A comparable situation associated with poverty and conflict may now be unfolding in the MENA. Our previous analysis showed a surprisingly high burden of NTDs disproportionately affecting an estimated 65 million people who live in extreme poverty in this region [4]. Shown in Table 1 is a reassessment of the NTD burden in the MENA based on newly released World Health Organization (WHO) and other estimates [5–13]. In total, approximately 50 million people in the MENA suffer from an NTD.

**Table 1:** The major neglected tropical diseases (NTDs) of the Middle East and North Africa (MENA).^a^

Disease	Number of cases	Comments and Reference
Ascariasis	24.3 million	Total infected population [5]
Schistosomiasis	9.2 million	Population requiring preventive chemotherapy for schistosomiasis annually in WHO Eastern Mediterranean region, 2013, minus Somalia and Sudan (which are not ordinarily considered MENA countries) [6]
Trichuriasis	8.7 million	Total infected population [5]
Hookworm	4.6 million	Total infected population [5]
Dengue	2.0 million	Total “apparent” cases in Egypt, Oman, Saudi Arabia, Syria, and Yemen [7]
Lymphatic filariasis (LF)	550,172	Population requiring preventive chemotherapy for LF in WHO Eastern Mediterranean region, 2013, minus Somalia and Sudan (which are not ordinarily considered MENA countries) [8]
Cutaneous leishmaniasis	465,700 to 810,000	Total incidence of the combined regions designated as Mediterranean and Middle East to Central Asia [9]
Malaria	107,267	Total confirmed cases in 2013 in WHO Eastern Mediterranean region endemic countries reporting: Algeria, Iran, Saudi Arabia, Yemen [10]
Visceral leishmaniasis	6,200 to 12,000	Total Incidence of the combined regions designated as Mediterranean and Middle East to Central Asia [9]
MERS	>1,000	Laboratory-confirmed cases [11]
Leprosy	<1,000	Registered cases in WHO Eastern Mediterranean region 2013 minus Pakistan, Somalia, and Sudan [12]
Trachoma	Endemic	Reported as “endemic” in Egypt, Iraq, Libya, Yemen [13]

^a^The MENA region typically includes the following countries: Algeria, Bahrain, Djibouti, Egypt, Iran, Iraq, Israel, Jordan, Kuwait, Lebanon, Libya, Malta, Morocco, Oman, Qatar, Saudi Arabia, Syria, Tunisia, United Arab Emirates, West Bank and Gaza, and Yemen.

Moreover, several important infections, including NTDs, have re-emerged in war-torn areas of Syria, such as cutaneous leishmaniasis (CL), scabies, hepatitis A, measles, rabies, polio, and tuberculosis, and in Afghanistan, where CL is now an important public health threat [14,15]. Social disruptions in Egypt may have helped to promote the emergence of dengue, together with the fact that the largest numbers of impoverished people from the MENA live in Egypt [4]. Additionally, even prior to the current outbreak of hostilities, Yemen had the single highest poverty rate in the MENA and suffered from multiple NTDs, including much of the schistosomiasis in the region [4].

**Fig 1:**
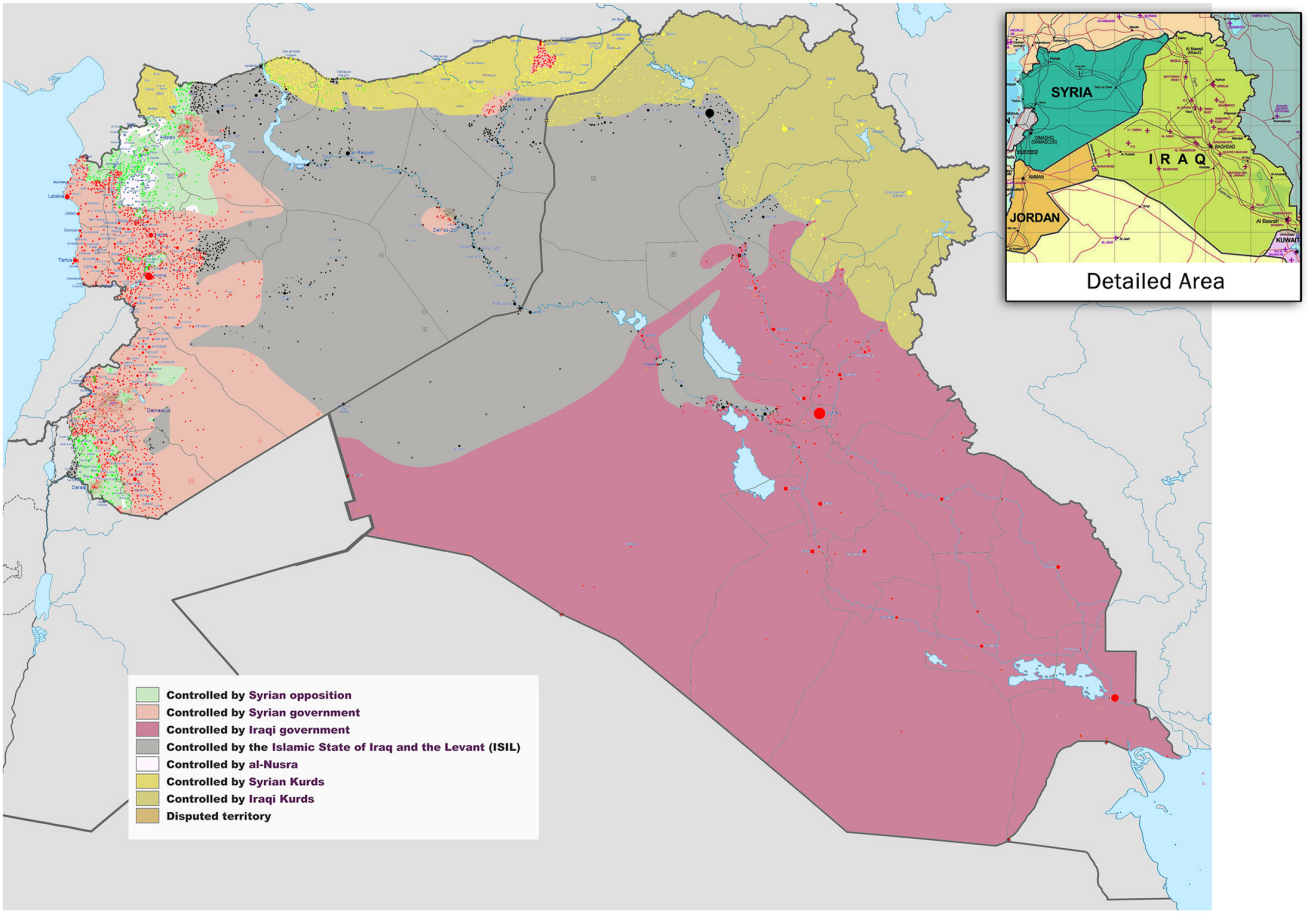
Syria and Iraq 2014-onward war map—occupied territories. By Wikimedia Commons user BlueHypercane761. Available here: https://commons.wikimedia.org/wiki/File:Syria_and_Iraq_2014-onward_War_map.png.

Superimposed on an already worrisome threat from tropical infections are the recent advances of the Islamic State (IS) in Iraq, Syria, and Libya, and Yemen (Fig 1). Currently the IS of Iraq and al-Sham (ISIS) is considered a pseudo-state with an estimated 30,000 fighters, together with a sophisticated command structure but with minimal to nonexistent health systems [16], while according to a recent report, Yemen’s health system is “moving from a crisis to a disaster” [17].

In this setting we must again anticipate breakdowns in public health infrastructure and, with those breakdowns, a steep rise in existing infections or the emergence of new ones. They could include many of the NTDs highlighted above, such as CL (or even visceral leishmaniasis), dengue, and scabies, as well as tuberculosis, hepatitis A and B, and classical childhood viral infections such as measles and polio [15]. Also of great concern is the emergence of zoonotic infections linked to animal trafficking across fractured international borders, such as brucellosis [14,18]. As another example, Middle Eastern Respiratory Syndrome (MERS)—recently appearing in Saudi Arabia and elsewhere on the Arabian Peninsula—could spread undetected through camel animal reservoirs.

## The Global Health Security Agenda (GHSA)

When Ebola virus emerged in West Africa in 2014, the global health community was slow in mounting a coordinated international response. To avoid a repeat, is there a possibility of mobilizing resources now in order to combat the NTDs and other emerging infections that will surely arise in the MENA? Anticipatory action plans could include expansion of public health emergency preparedness through the United States—launched Global Health Security Agenda (GHSA) in partnership with the World Health Organization (WHO), the Food and Agriculture Organization (FAO), and the World Organisation for Animal Health (OIE) [19]. Launched in February 2014, the GHSA has an overarching mission to prevent and reduce the likelihood of infectious disease outbreaks by detecting threats early and providing for rapid and multisectoral responses [19]. GHSA also furthers progress towards full implementation of WHO International Health Regulations 2005 (IHR).

Among the key components of the GHSA are immunizations and access to vaccines. As an example, in 2013 when poliovirus was detected in Syria from Pakistan, the MENA responded with a robust vaccination initiative [20], while UNICEF has since set ambitious goals for vaccinating refugees [21]. It is unlikely that today such responses would be possible in IS-held conflict areas [14]. However, it remains highly desirable to consider the possibility of implementing cease-fires for the purpose of vaccination in IS-occupied zones, similar to those conducted previously in Sudan and other war-torn areas [22].

## New Vaccines for the MENA

But what if diseases emerge across the MENA region for which vaccines and other countermeasures have not yet been developed? Given that a number of diseases might be primarily of regional instead of global importance, it is unlikely they will be targeted for vaccine development by the multinational pharmaceutical companies. We saw this situation previously when Ebola virus vaccines were not available throughout all of 2014, and these vaccines are only now entering phase 1 trials, more than a year after the epidemic ignited in West Africa.

We need new vaccines developed, tested, and possibly stockpiled for the MENA region [23,24]. These might include vaccines to prevent NTDs such as leishmaniasis, but also MERS and other emerging viral infections. While currently there are minimal capabilities for developing new neglected and emerging disease vaccines in MENA, several MENA nations are potentially well placed to initiate such activities. Such programs could be an important first step in creating regional leadership in this vital area.

In a June 2009 speech in Cairo, Egypt, President Barack Obama launched an initiative known as “New Beginning” in order to engage the Muslim world in science and technology diplomacy, with an emphasis on agriculture, climate, energy, water, the environment, and sustainability [23]. A key element of the outreach is a US Science Envoy program to send notable American scientists to Organisation of Islamic Cooperation (OIC) countries to promote scientific collaborations.

Given the urgent need to develop and possibly stockpile new vaccines for the major NTDs emerging out of MENA, there are opportunities for “vaccine science diplomacy,” analogous to events of the post-Sputnik era of the Cold War, when an American—Soviet collaboration led to final development and testing of the Sabin polio vaccine [23,24].

As noted above, there is a high likelihood that vaccines of regional importance will not be developed, tested, or prioritized by any of the major multinational pharmaceutical companies, and currently there is minimal capacity for developing new human vaccines in MENA. As a potential alternative, there are approximately a half-dozen international non-profit vaccine product development partnerships (PDPs), which use industry practices to develop new vaccines for HIV/AIDS, tuberculosis, malaria, and NTDs [21]. The PDPs in turn work with member organizations of the Developing Country Vaccine Manufacturers Network (DCVMN), which for the MENA, might include fill and finish facilities for imported bulk vaccines, currently located in Egypt, Saudi Arabia, and elsewhere [25].

The Sabin Vaccine Institute and Texas Children’s Hospital Center for Vaccine Development is a Houston-based PDP (Sabin PDP) currently developing vaccines for many of the NTDs affecting MENA and other areas of poverty in developing countries. Among the potential target countries for building vaccine-development capacity through international scientific collaboration are OIC nations such as Morocco, Tunisia, Saudi Arabia, and Qatar.

### Morocco and Tunisia

Morocco ranks 129 out of 187 in terms of its human development index and is categorized by the United Nations Development Programme as a medium development country [26]. Despite a significant level of extreme poverty, the country was one of the first to achieve the elimination of trachoma, a major NTD. However, according to some published reports and databases, Morocco may also have the third largest number of cases of cutaneous leishmaniasis in MENA, and the largest number of trichuriasis (whipworm) cases [4]. Morocco aspires to become a leader in technology innovation for Africa, and in so doing, could join other so-called “innovative developing countries” (IDCs), loosely defined as nations that are unexpectedly high achievers in terms of their ability to innovate and produce important technologies [27]. Institutions such as Morocco’s Institut Pasteur in Casablanca and their National Institute of Hygiene in Rabat, the capital, or one or more of their universities (including a rapidly growing university in Fez), together with a robust private-sector pharmaceutical industry, could expand their nascent vaccine-development capabilities jointly with the Sabin PDP. Similarly, in Tunisia, the Institut Pasteur in Tunis has some vaccine-development capabilities, especially for Bacillus Calmette—Guérin and therapeutic sera [28].

### Saudi Arabia

**Fig 2:**
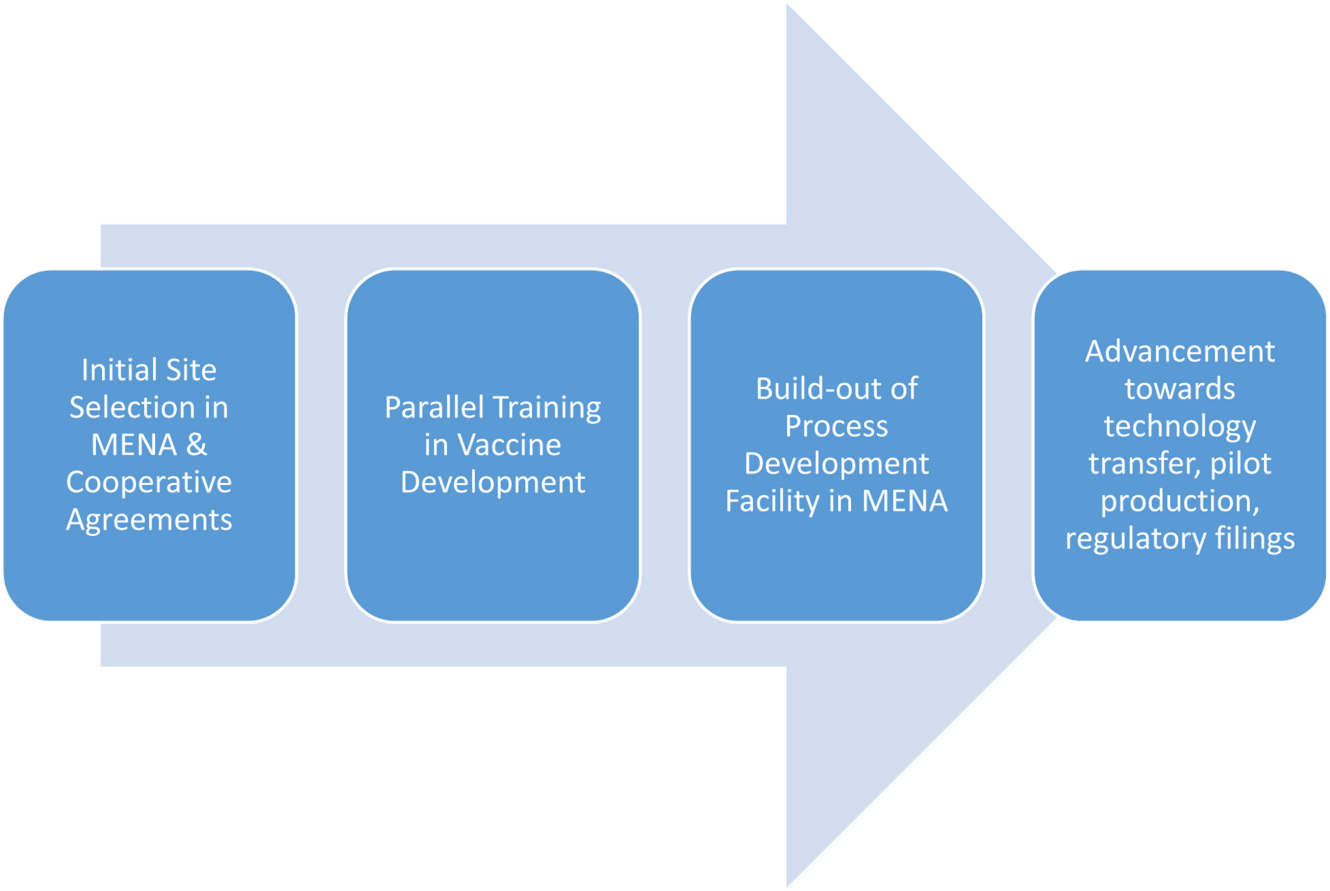
Steps in the development cycle for implementing vaccine science diplomacy in the MENA.

The Kingdom of Saudi Arabia has invested heavily to establish world-class research universities institutions, including some Saudi universities now ranked among the highest in the MENA region according to *US News and World Report* rankings [29]. These include King Saud University, Alfaisal University, and King Faisal Specialist Hospital and Research Centre, located in Riyadh; and in the Eastern Province, University of Dammam, King Fahd University of Petroleum and Minerals, and Johns Hopkins Aramco Healthcare. King Abdullah University of Science and Technology in Thuwal and King Abdulaziz University in Jeddah are also leading universities, while the University of Jazan near the border with Yemen also has experience in tropical diseases. Such facilities at national universities or institutes could be expanded in order to build early-stage vaccine-development capabilities that match those of the Sabin PDP. An emphasis would be put on process development—developing reproducible processes for pilot-scale manufacture under strict quality control and quality assurance. Success on this front will also depend on advanced training in each step of the vaccine development cycle, both in Houston and in the designated MENA nation (Fig 2). The major deliverables of such vaccine science diplomacy activities would include the development and clinical testing of a vaccine that targets an NTD of national or regional importance. These activities would also expand Saudi Arabia’s biotechnology capacity. Such a vaccine development initiative could interface with Arabio, the Gulf’s first biopharmaceutical company, with capabilities for final production of diphtheria, tetanus, pertussis, and other vaccines [30]

### Qatar, Kuwait, United Arab Emirates, and Egypt

The other Gulf monarchies also have great potential for hosting vaccine development infrastructure. For instance, preeminent Qatari institutions are located in their Education City, an initiative of the Qatar Foundation for Education, Science and Community Development. Egypt also has many of the top-ranked universities in the MENA region.

As an example of an ongoing international collaboration, our Sabin PDP works together with the University of Malaya to build out neglected disease vaccine-development capacity. Over a three-year period, Malaysian scientists are working in our Houston-based laboratories to learn each step of the vaccine development cycle, while working towards building a parallel vaccine process development or pilot facility in Kuala Lumpur. This facility could lead to future neglected vaccines suitable for early-stage clinical testing.

Ultimately, halal vaccine development production by institutions based in an OIC country, such as those highlighted above, could help to facilitate vaccine uptake in conflict and post-conflict areas, such as Pakistan and northern Nigeria.

## Next Steps and Concluding Remarks

According to former US Secretary of State Henry Kissinger, “science and technology are the governing concepts of our age” and ones that are replacing nationalism as a major “leitmotif” of the 21st century [31]. While pockets of science and technology have advanced in the MENA region, overall vaccine biotechnology has lagged. In terms of manufacturers that produce vaccines accessible for developing countries and belong to the DCVMN, only four come from the MENA region—one each in Saudi Arabia (AraBio) and Egypt (Vacsera), and two in Iran (Institut Pasteur and Razi Vaccine and Serum Research Institute) [23–25], and none produces vaccines that are pre-qualified by the WHO for export [32]. Instead, the region mostly imports its vaccines or vials in bulk [14]. As a consequence, the MENA region is highly vulnerable to regional neglected and emerging infections, including MERS, schistosomiasis, leishmaniasis, scabies, and other NTDs, which may not be targeted by multinational pharmaceutical companies.

Vaccine-development capability could become an important global health and security theme for the coming decade. It is an area that builds on a growing, robust, and more comprehensive GHSA [19] and that promotes the WHO IHR. There is an opportunity to build infrastructure for indigenous vaccine production in the MENA region in anticipation of new diseases emerging from poverty and conflict. The US could assist in promoting vaccine development in the countries highlighted above, where there are already existing elements of political and economic security and strengths in biotechnology. Thus, in addition to the key role of US and international PDPs described here, the US government could also enhance vaccine development activities in the GHSA by incorporating and augmenting resources and talent through its Departments of State (e.g., Office of Global Health Diplomacy and US Agency for International Development), Health and Human Services (e.g., National Institutes of Health and Office of Global Affairs), and Defense (e.g., Walter Reed Army Institute of Research), among others.

Collaboration, both regionally and with the broader international community, to enhance capacity in the MENA to develop vaccines is an important means to develop sustainable health system structures to support health resilience. Collaboration between the US and the international community on vaccines is already a key element of the GHSA; we should maintain momentum and look for new, willing partners in these efforts. Ultimately, expanding preparedness and vaccine-development capacity in the MENA could help to avert an international public health crisis such as the 2014 Ebola virus outbreak and save thousands of lives in the region.
